# Factor Structure and Factorial Invariance of the Strengths and Difficulties Questionnaire among Children of Prisoners and their Parents

**DOI:** 10.1007/s12187-017-9464-9

**Published:** 2017-03-24

**Authors:** Kathryn Sharratt, Daniel Boduszek, Bernard Gallagher, Adele Jones

**Affiliations:** School of Human & Health Sciences, University of Huddersfield, Queensgate, Huddersfield, HD1 4DH UK

**Keywords:** Strengths and difficulties questionnaire (SDQ), Children of prisoners, Confirmatory factor analysis, Bifactor modelling, Factorial invariance

## Abstract

Parental imprisonment has been linked to a variety of adverse psychological outcomes for children and adolescents. The Strengths and Difficulties Questionnaire (SDQ) has been widely used to assess behavioural and emotional difficulties among 7–17 year olds in the general population and more recently has been utilised among samples of children of prisoners. Previous research has variously tested traditional one-, three- and five- factor solutions to the SDQ, and more recently one bifactor solution has been examined. Based on a sample of children of prisoners (*N* = 724) and their non-imprisoned parent or caregiver (*N* = 658), the aim of the present study was to simultaneously compare nine alternative factor structures, including previously tested models and alternative bifactor solutions. Tests of factorial invariance and composite reliability were also performed. The five-factor model was found to provide the best fit for the data. Tests of factorial invariance revealed that the five-factor model provided an equally acceptable, but not identical fit, among boys and girls. Composite reliability scores were low for the Conduct Problems and Peer Problems subscales. The utility of the SDQ in measuring psychological functioning in response to parental imprisonment is discussed.

## Introduction

In recent years, increased empirical attention has been paid to the adverse psychological consequences of parental imprisonment for children and adolescents (e.g. Bocknek et al. 2009; Hissel et al. 2011). Emerging evidence suggests that boys affected by parental imprisonment experience more externalising problems whereas girls are more susceptible to internalising problems (Murray et al. 2009; Murray and Farrington 2008). In order to further our understanding of the consequences of parental imprisonment, we need to identify instruments that provide a reliable and valid assessment of behavioural and emotional outcomes among this group. The desire to compare the psychological adjustment of boys and girls also underscores the importance of examining factorial invariance.

The Strengths and Difficulties Questionnaire (SDQ; Goodman 1997) comprises of five subscales relevant to measuring internalising and externalising problems, and represents a potentially useful instrument for understanding the experiences of children of prisoners. The five subscales are Emotional Problems (EP); Peer Problems (PP); Conduct Problems (CP); Hyperactivity (H) and Prosocial Behaviour (PS). The first four subscales can be summed to provide a Total Difficulties Score (TDS), whereas the PS subscale assesses strengths and is considered independent of the difficulties subscales. The SDQ has been widely utilised within the general population and clinical settings, and more recently has been administered to children of prisoners (Lösel et al. 2012). The availability of parallel versions of the form for completion by self-report, parents and teachers is considered advantageous in terms of facilitating the triangulation of results (Goodman 1997).

Although the SDQ is highly regarded for its clinical utility (e.g. Becker et al. 2004; Muris et al. 2003), evidence concerning the psychometric validity of the measure is less convincing. Several studies have reported low internal consistencies for the SDQ, particularly for the CP and PP subscales (e.g. Di Riso et al. 2010; McCrory and Layte 2012). Exploratory factor analysis (EFA) has supported the existence of five separate subscales in various samples across Europe (e.g. Smedje et al. 1999; Muris et al. 2003; Capron et al. 2007). However, some investigators did observe a tendency for the positively worded items to load onto the same subscale suggesting the possibility of a method effect (e.g. Muris et al. 2003), and others observed subtle differences in the factor loadings among boys and girls (e.g. Smedje et al. 1999).

In contrast to EFA, confirmatory factor analysis (CFA) provides a robust indication of whether items load onto latent constructs in the anticipated manner in the absence of measurement error (Bollen 1989). The majority of studies have confirmed that the five-factor model provides an adequate fit for the self-report, parent and teacher versions of the scale (e.g. d’Acremont and Van der Linden 2008; Stone et al. 2013; Van Roy et al. 2008). In support of the existence of a method factor, some CFAs have found that the inclusion of a separate factor for the positively worded items substantially improved the fit of the five-factor model (McCrory and Layte 2012; Palmieri and Smith 2007). Although few of these studies tested for factorial invariance, concerns about the SDQ performing unequally among boys and girls have not been supported (d’Acremont and Van der Linden 2008; Stone et al. 2013).

A small number of CFAs have provided evidence for alternative, but still theoretically justifiable, model conceptualisations. Retaining the PS factor, but replacing the EP and PP subscales, and CP and H subscales, with internalising and externalising factors respectively, was found to provide adequate model fit in Italy and Belgium (Di Riso et al. 2010; Van Leeuwen et al. 2006). In the UK, Goodman et al. (2010) found little support for replacing the subscales; instead a model with higher-order internalizing and externalizing factors achieved acceptable model fit values.

In the only study to test a bifactor model, Kobór et al. (2013), found support for a general “total difficulties” factor and five grouping factors akin to the original subscales. However, this solution was theoretically incompatible with Goodman’s (1997) original conceptualisation of the scale as the general factor incorporated not only the four difficulties subscales but also the positive PS subscale.

In summary, previous studies have most frequently found support for a five-factor solution but alternative model structures have been validated including a three-factor and bifactor model. Whereas previous research has typically examined between one and four models, the aim of the present study was to *simultaneously compare* the fit of nine alternative factor structures, including alternative bifactor solutions. Analysis was based on a European sample of children of prisoners and their non-imprisoned parents or caregivers. As noted above, the SDQ is theoretically relevant to understanding the outcomes of parental imprisonment, and indeed has already been administered to such a sample. Given the desire to compare outcomes for boys and girls affected by parental imprisonment, but sparse evidence concerning factorial invariance of the SDQ, this paper also presents tests of factorial invariance.

## Materials and Method

### Participants

Participants were children with a parent/caregiver in prison (*N* = 724) and their non-imprisoned parent/caregiver (*N* = 658). Participants were recruited by non-governmental organisations as part of their normal work at prison visitor centres and counselling centres in the UK (N children =273; N parent =221), Germany (N children =145; N parent =144), Romania (N children =246; N parent =247) and Sweden (N children =60; N parent =46). The sample consisted of similar proportions of boys and girls (54.28% and 45.72% respectively). Children were aged between 7 and 17 years and had a mean age of 11.27 years (SD = 3.12). Younger children (aged 7–12 years; *N* = 502) accounted for a larger proportion of the sample than older children (aged 13–17 years, *N* = 222). Mothers accounted for the largest proportion of parents/caregivers (73.3%; M age = 39.02, SD = 9.53). Data on ethnicity was only available for the UK and Romania, where the majority of children (86.81%) and parents/caregivers (88.68%) were White.

### Measure

The self-report and parent version of the SDQ comprise of 25 parallel items which are responded to on a 3-point scale (0 = not true; 1 = somewhat true; 2 = certainly true). The questionnaire consists of five subscales measuring Emotional Problems (EP), Peer Problems (PP), Conduct Problems (CP), Hyperactivity (H) and Prosocial Behaviour (PS). The previously validated translations of the scale used in this study, and details on the scoring procedures, can be found at: http://sdqinfo.org.

### Analysis

The dimensionality of the SDQ was investigated using confirmatory factor analytic (CFA) techniques with robust maximum likelihood (MLR) estimation in Mplus version 6.0 (Muthen and Muthen 1998–2010). Nine alternative model conceptualisations were specified and tested, making this the most comprehensive examination of the SDQ factor structure to date. The first-order conceptualisations were (i) a 25-item unidimensional model; (ii) a model comprising of internalising, externalising and prosocial factors; and (iii) a model with five-factors reflecting the original subscales. Three bifactor models were also tested in which items were allowed to load onto the hypothesised subscale and one grouping or method factor (see Reise et al. 2010). These models were (ii) five grouping factors representing the original subscales and one general “total difficulties” factor for the four difficulties subscales; (i) five grouping factors and two general factors representing internalising and externalising problems; and (iii) five grouping factors and one method factor representing the positively worded items. Finally, the three hierarchical models were (i) a five factor model with a higher order factor underlying all 25 items; (ii) a five-factor model with a higher order “total difficulties” factor and a separate PS factor; and (iii) a five-factor model with two higher order internalising and externalising factors and a separate PS factor. In all cases measurement error terms remained uncorrelated as suggested in previous research (see Boduszek et al. 2013).

Overall model fit was assessed using a range of goodness-of-fit statistics and the appropriateness of the model parameters. The chi-square (*χ2*) statistic assessed the sample and implied covariance matrix; a good fitting model is indicated by a non-significant result. The chi-square statistic is, however, strongly associated with sample size, and as such good models tend to be over-rejected. The Comparative Fit Index (CFI; Bentler 1990) and the Tucker Lewis Index (TLI; Tucker and Lewis 1973) are measures of how much better the model fits the data compared to a baseline model where all variables are uncorrelated. For these indices values above .95 indicate good model fit but values above .9 are considered acceptable (Bentler 1990; Hu and Bentler 1999). The standardized root mean-square residual (SRMR; Joreskog and Sorbom 1981) and the root mean-square error of approximation (RMSEA; Steiger 1990) are also presented. Ideally these indices should be less than .05, but values less than .08 are considered acceptable (Bentler 1990; Hu and Bentler 1999). The Akaike Information Criterion (AIC; Akaike 1974) was used to compare the alternative models, with the smaller value indicating the best fitting model.

## Results

Descriptive statistics, including measures of internal consistency, are presented in Tables 1 and 2. Traditional measures such as Cronbach’s alpha have been criticised within a latent variable modelling context given the propensity to over- or under-estimate scale reliability (see Raykov 1998). In order to provide a more rigorous assessment of internal reliability, the current study calculated composite reliability using the formula: *CR* = (sum of *λ*_i_)^2^/(sum of *λ*_i_)^2^ + (sum of *θ*_i_), where *CR* = reliability of the factor score, *λ*_i_ = standardized factor loading, and *θ*_i_ = standardised error variance. Values greater than .60 are generally considered acceptable (Bagozzi and Yi 1988; Diamantopoulos and Siguaw 2000). Composite reliability was low for all children (total sample, boys only, and girls only) and parents on the PP subscale, and for the total sample, and in particular girls, on the CP subscale.

**Table 1:** Descriptive statistics and internal consistency for self-report and parent versions of the SDQ

	Children (total sample)				Girls				Boys				Parents			
	M	SD	α	CR	M	SD	α	CR	M	SD	α	CR	M	SD	α	CR
Emotional Problems	3.363	2.474	.697	.703	3.612	2.479	.691	.698	3.212	2.461	.702	.711	3.539	2.643	.756	.757
Conduct Problems	2.451	1.977	.585	.587	2.045	1.671	.474	.467	2.793	2.155	.625	.626	2.616	2.173	.670	.674
Hyperactivity	4.047	2.391	.663	.662	3.699	2.231	.615	.617	4.328	2.492	.688	.683	4.301	2.502	.696	.693
Peer Problems	2.441	1.749	.380	.442	2.345	1.734	.409	.460	2.518	1.761	.354	.390	2.558	1.915	.511	.501
Prosocial Behaviour	8.154	1.924	.729	.730	8.486	1.754	.671	.675	7.874	2.023	.757	.754	7.848	2.021	.712	.713
Total Difficulties	12.259	6.115	.768	.859	11.603	5.785	.686	.841	12.792	6.354	.775	.865	12.958	6.920	.837	.888

**Table 2:** Descriptive statistics and for younger and older children

	Children aged 7–12 years		Children aged13–17 years	
	M	SD	M	SD
Emotional Problems	3.334	2.423	3.439	2.524
Conduct Problems	2.535	2.121	2.734	2.282
Hyperactivity	4.083	2.354	3.926	2.473
Peer Problems	2.500	1.756	2.280	1.745
Prosocial Behaviour	8.329	1.868	7.861	1.991
Total Difficulties	12.554	5.811	12.458	6.078

There was a moderate positive correlation between the scores provided by boys only and parents on the EP (.57), PP (.48), CP (.60), H (.56) and PS (.49) subscales. Correlations between girls only and parents were somewhat weaker on the EP (.47), PP (.39), CP (.40), H (.48) and PS (.45) subscales. All correlations were statistically significant at *p* < 0.001.

### Testing Competing Models

Table 3 reports the fit indices and comparative fit indices for the nine alternative models of the SDQ in children (total sample, girls only, and boys only) and parents. None of the models provided a good approximation of the data based on CFI and TLI statistics.

**Table 3:** Fit indices for nine alternative models of SDQ

Models	χ2 (df)	CFI/TLI	SRMR	RMSEA	AIC
Children (total sample)					
1 factor^a^	2063.55 (275)	.408/.354	.111	.096	33,289.58
3 factors^b^	1147.53 (272)	.710/.680	.090	.067	32,397.59
5 factors^c^	1044.07 (265)	.743/.708	.086	.064	32,305.01
Bifactor (5 grouping +1 general)	1082.77 (250)	.724/.669	.099	.069	32,316.49
Bifactor (5 grouping +2 general)	1883.50 (261)	.463/.383	.264	.098	33,278.94
Bifactor (5 grouping +1 method)	1343.17 (270)	.645/.605	.135	.075	32,621.13
5 factors +1 hierarchical	1232.29 (270)	.682/.646	.095	.071	32,470.52
5 factors +1 hierarchical with separate PS^d^	1268.89 (271)	.670/.634	.104	.072	32,520.87
5 factors +2 hierarchical with separate PS^e^	1193.84 (270)	.694/.660	.102	.070	32,450.38
Girls					
1 factor	990.51 (275)	.412/.358	.104	.089	14,746.34
3 factors	707.34 (272)	.642/.605	.090	.070	14,477.19
5 factors	638.93 (265)	.692/.652	.085	.066	14,418.20
Bifactor (5 grouping +1 general)	653.20 (250)	.668/.602	.090	.070	14,428.46
Bifactor (5 grouping +2 general)	747.23 (259)	.598/.535	.128	.076	14,576.71
Bifactor (5 grouping +1 method)	867.92 (270)	.583/.537	.129	.083	14,562.01
5 factors +1 hierarchical	714.97 (270)	.634/.593	.091	.071	14,479.18
5 factors +1 hierarchical with separate PS	728.90 (271)	.623/.583	.100	.072	14,502.69
5 factors +2 hierarchical with separate PS	690.51 (268)	.652/.611	.088	.070	14,456.80
Boys					
1 factor	1410.58 (275)	.394/.339	.124	.104	18,241.70
3 factors	843.94 (272)	.695/.663	.101	.074	17,669.03
5 factors	789.49 (265)	.721/.684	.099	.072	17,628.75
Bifactor (5 grouping +1 general)	951.59 (250)	.627/.554	.282	.086	17,896.53
Bifactor (5 grouping +2 general)	857.40 (259)	.682/.631	.121	.078	17,727.94
Bifactor (5 grouping +1 method)	1073.62 (270)	.634/.593	.146	.089	17,800.21
5 factors +1 hierarchical	923.31 (270)	.651/.613	.108	.080	17,735.76
5 factors +1 hierarchical with separate PS	920.70 (271)	.653/.616	.115	.080	17,746.46
5 factors +2 hierarchical with separate PS	810.71 (268)	.710/.676	.101	.073	17,632.94
Parents (total sample)					
1 factor	1562.58 (275)	.580/.542	.096	.085	30,645.60
3 factors	948.78 (272)	.778/.755	.077	.062	29,915.38
5 factors	861.19 (265)	.825/.780	.072	.055	29,724.58
Bifactor (5 grouping +1 general)	811.16 (250)	.814/.777	.076	.059	29,783.36
Bifactor (5 grouping +2 general)	1072.55 (259)	.735/.693	.133	.070	30,106.13
Bifactor (5 grouping +1 method)	1373.95 (270)	.640/.600	.155	.080	30,404.03
5 factors +1 hierarchical	971.05 (270)	.770/.745	.079	.064	29,935.71
5 factors +1 hierarchical with separate PS	1065.50 (271)	.740/.712	.104	.068	30,042.99
5 factors +2 hierarchical with separate PS	885.14 (268)	.798/.774	.073	.060	29,840.21

^a^Glenn et al. (2013)

^b^Di Riso et al. (2010), Van Leeuwen et al. (2006)

^c^Stone et al. (2013), Van Roy et al. (2008)

^d^McCrory and Layte (2012)

^e^Goodman et al. (2010)

Based on RMSEA and SRMR the five-factor model of the SDQ was found to be an adequate representation of the data for all samples included in the study. Moreover, substantial improvements were observed in CFI and TLI for the five-factor model. This model which includes five correlated factors was determined the best approximation of the covariation matrix in the obtained data based upon all fit indices. This model also demonstrated a lower AIC value than the alternative models further indicating its statistical superiority.

The adequacy of this model can also be determined in relation to its parameter estimates. As can be seen in Table 4 all items displayed statistically significant (*p* < .001) factor loadings on respective latent factors. All factor loadings were in the expected direction and exceeded 0.4 with the exception of items 7, 10, 14, 16 and 18 in the children samples and items 17 and 20 in the parents sample.

**Table 4:** Standardized factor loadings for five factor SDQ model

Items	Children	Girls	Boys	Parents
Emotional Problems				
1.I get a lot of headaches, stomach-aches or sickness	.493	.511	.476	.533
2.I worry a lot	.518	.599	.456	.594
3.I am often unhappy, down-hearted or tearful	.732	.676	.771	.738
4.I am nervous in new situations, I easily lose confidence	.492	.464	.521	.616
5.I have many fears, I am easily scared	.589	.552	.630	.612
Conduct Problems				
6.I get very angry and often lose my temper	.636	.552	.684	.596
7.I usually do as I am told	.294	.269	.288	.470
8.I fight a lot. I can make other people do what I want	.505	.356	.560	.608
9.I am often accused of lying or cheating	.540	.483	.567	.583
10.I take things that are not mine from home, school or elsewhere	.358	.257	.378	.439
Hyperactivity				
11.I am restless, I cannot stay still for long	.640	.508	.707	.645
12.I am constantly fidgeting or squirming	.655	.555	.718	.662
13.I am easily distracted, I find it difficult to concentrate	.565	.541	.558	.595
14.I think before I do things	.367	.375	.364	.430
15.I finish the work I am doing, my attention is good	.408	.484	.364	.443
Peer Problems				
16.I am usually on my own, I generally play alone or keep to myself	.386	.405	.359	.498
17.I have one good friend or more	.322	.377	.279	.351
18.Other people my age generally like me	.383	.462	.270	.418
19.Other children or young people pick on me or bully me	.498	.497	.513	.534
20.I get on better with adults than with people my own age	.195	.152	.256	.231
Prosocial Behaviour				
21.I try to be nice to other people, I care about their feelings	.626	.538	.648	.641
22.I usually share with others (food, games, pens etc.)	.476	.427	.504	.512
23.I am helpful is someone is hurt, upset or feeling ill	.643	.624	.672	.597
24.I am kind to younger children	.611	.540	.635	.530
25.I often volunteer to help others (parents, teachers, other children)	.600	.576	.617	.597

All factor loading are significant at *p* < .001

### Model Invariance for Boys and Girls

Tests of factorial invariance were conducted between boys (*n* = 393) and girls (*n* = 331) using the five-factor model as the baseline model. Following the procedure of Bollen (1989), a hierarchy of increasingly restrictive models were specified and tested. Firstly, the model parameters were constrained to be equal between boys and girls to determine if that model performed less well than one that was unconstrained (configural model). This test of invariance of form, or that this five-factor model held in both samples, was poor but acceptable based on RMSEA and SRMR statistics (χ2 (575) = 1565.44, *p* < .05; RMSEA = .070 [95%CI = .066/.074]; CFI = .680; TLI = .666; SRMR = .090); as were the tests of equal factor loadings (χ2 (550) = 1477.67, *p* < .05; RMSEA = .069 [95%CI = .065/.073]; CFI = .700; TLI = .673; SRMR = .090); and equal factor variances/covariances (χ2 (590) = 1596.30, *p* < .05; RMSEA = .070 [95%CI = .066/.074]; CFI = .674; TLI = .669; SRMR = .096). Satorra-Bentler scaled χ2 difference tests (TRd), including the difference test scaling correction (CD) were computed to compare the model with equal factor loadings (CD = 0.997, TRd = 90.04, Δdf = 25, *p* < .05), and the model with equal factor variances/covariances (CD = 1.225, TRd = 31.79, Δdf = 15, *p* < .05), to the configural model. In both cases a statistically significant difference was observed, suggesting a different pattern of item loadings and factor covariances among boys and girls. As can be seen in Table 3, nine items loaded more strongly onto the respective factors among boys (3, 6, 8, 10, 11, 12, 20, 21 and 24), and four items loaded more strongly among girls (2, 15, 17 and 18). Six of the factor covariances were moderate-strong in girls (0.51–0.84) compared to three in boys (0.63–0.98), but the most apparent discrepancy was that the EP and CP factors correlated positively among girls (0.65) and negatively among boys (−0.43). Tests of factorial variance were not conducted for the parent data due to an insufficient number of male parents in the sample.

## Discussion

The aim of this study was to examine the factor structure and factorial invariance of the SDQ within a sample of children of prisoners and their non-imprisoned parents/caregivers. Traditional CFA and bifactor modelling techniques were utilised to comprehensively compare the fit of nine alternative model structures. On the basis of a range of goodness-of-fit statistics, the five-factor model was considered to provide the best fit for the self-report and parent data. This finding supports Goodman’s (1997) original conceptualisation of the SDQ, and also previous factor analyses (e.g. Capron et al. 2007; Stone et al. 2013). Model fit indices were comparatively poorer for alternative three-factor and bifactor models validated elsewhere in the literature (e.g. Di Riso et al. 2010; Goodman et al. 2010; Kobór et al. 2013), and the inclusion of a positive-item method factor failed to improve model fit (as in McCrory and Layte 2012; and Palmieri and Smith 2007).

The five-factor model, however, was only acceptable according to two out of the four overall model fit indices. Further inspection of the item loadings also revealed that a number of the values were unacceptably low, especially for the CP and PP subscales. Problems with the CP and PP subscales were further reflected in low composite reliability scores for these subscales. This might reflect the fact that the subscales contain only five items or might be indicative of the subscales measuring more heterogeneous constructs that intended. This finding is consistent with previous literature within the general population (e.g. Di Riso et al. 2010; McCrory and Layte 2012), and indicates that these subscales in particular should be interpreted with caution by researchers and clinicians. Qualitative research examining the meaning of items on these subscales might be beneficial in terms of informing the potential reorganisation or rephrasing of items to better capture the intended latent constructs and therefore improve model fit and internal consistency.

Tests of factorial invariance revealed that the five-factor model provided an equally acceptable but not identical fit for boys and girls. This means that gender differences in subscale means or correlations between subscales (either on the SDQ or with other instruments) might be reflective of differences in the meaning of items for boys and girls rather than genuine differences in emotional and behavioural outcomes. With regards to furthering our understanding of the differential impact of parental imprisonment on girls and boys (see Murray et al. 2009; and Murray and Farrington 2008), these findings suggest that the SDQ might not be the most appropriate instrument in its present format. Again, qualitative research might be advantageous in terms of understanding the meaning of items for boys and girls.

As outlined in previous literature, children of prisoners are at increased risk of offending behaviour and mental health problems (Murray et al. 2009; Murray and Farrington 2008), and therefore it is perhaps not surprising that they are disproportionately represented in clinical populations (Phillips et al. 2002). Screening children of prisoners might offer the opportunity to provide early intervention and prevention of later problems. The SDQ is frequently used for screening purposes in clinical settings and it is plausible that children of prisoners might complete the instrument during their contact with metal health services. A robust factor structure is crucial to understanding the nature of the difficulties experienced and the aspects of psychological functioning that should be prioritised for intervention, as well as informing the content of interventions. Given the findings of this study, it is suggested that the SDQ might suffice as a preliminary assessment of psychological functioning but more in-depth assessments with successfully validated instruments are warranted.

To date, the SDQ has primarily been used for screening purposes rather than to measure changes in psychological functioning or evaluate treatment outcomes. Test-retest reliability is subject to developmental changes, environmental factors but also the stability of factor structures. Given the findings of the present study, it is recommended that any clinicians or researchers considering using the SDQ to measure changes in psychological functioning first examine the test-retest reliability of the instrument within their sample.

Although this paper has provided a more comprehensive assessment of the construct validity of the SDQ and has made a novel contribution to the literature surrounding the administration of the SDQ to children of prisoners, the study is not without its limitations. The modest sample size and homogeneity of the sample meant that further tests of factorial invariance could not be performed, for example according to the country of origin or the gender of the non-imprisoned parent.

In conclusion, the five-factor model only partially satisfied the criteria for acceptable model fit, but was superior to alternative conceptualisations. Item loadings and scores for internal consistency suggested that the CP and PP subscales were most problematic. Tests of factorial invariance also revealed that differences in model fit among boys and girls. Implications of these findings for research and clinical practice with children of prisoners were discussed.
